# Comparative analysis of IR-Biotyper, MLST, cgMLST, and WGS for clustering of vancomycin-resistant *Enterococcus faecium* in a neonatal intensive care unit

**DOI:** 10.1128/spectrum.04119-23

**Published:** 2024-03-05

**Authors:** Sunggyun Park, Namhee Ryoo

**Affiliations:** 1 Departments of Laboratory Medicine, Keimyung University School of Medicine, Daegu, South Korea; Foundation for Innovative New Diagnostics, Switzerland

**Keywords:** nosocomial outbreak, VREFM, IR-Biotyper

## Abstract

Healthcare-associated infections caused by vancomycin-resistant *Enterococcus faecium* (VREFM) pose a significant threat to healthcare. Confirming the relatedness of the bacterial isolates from different patients is challenging. We aimed to assess the efficacy of IR-Biotyper, multilocus sequencing typing (MLST), and core-genome MLST (cgMLST) in comparison with whole-genome sequencing (WGS) for outbreak confirmation in the neonatal intensive care unit (NICU). Twenty VREFM isolates from four neonates and ten control isolates from unrelated patients were analyzed. Genomic DNA extraction, MLST, cgMLST, and WGS were performed. An IR-Biotyper was used with colonies obtained after 24 h of incubation on tryptic soy agar supplemented with 5% sheep blood. The optimal clustering cutoff for the IR-Biotyper was determined by comparing the results with WGS. Clustering concordance was assessed using the adjusted Rand and Wallace indices. MLST and cgMLST identified sequence types (ST) and complex types (CT), revealing suspected outbreak isolates with a predominance of ST17 and CT6553, were confirmed by WGS. For the IR-Biotyper, the proposed optimal clustering cut-off range was 0.106–0.111. Despite lower within-run precision, of the IR-Biotyper, the clustering concordance with WGS was favorable, meeting the criteria for real-time screening. This study confirmed a nosocomial outbreak of VREFM in the NICU using an IR-Biotyper, showing promising results compared to MLST. Although within-run precision requires improvement, the IR-Biotyper demonstrated high discriminatory power and clustering concordance with WGS. These findings suggest its potential as a real-time screening tool for the detection of VREFM-related nosocomial outbreaks.

**IMPORTANCE:**

In this study, we evaluated the performance of the IR-Biotyper in detecting nosocomial outbreaks caused by vancomycin-resistant *Enterococcus faecium*, comparing it with MLST, cgMLST, and WGS. We proposed a cutoff that showed the highest concordance compared to WGS and assessed the within-run precision of the IR-Biotyper by evaluating the consistency in genetically identical strain when repeated in the same run.

## INTRODUCTION

In a confined space and within a limited timeframe, if the same strain, especially multidrug-resistant strains confirmed as pathogens, is identified from two or more patients, a nosocomial outbreak may be suspected (1). In a tertiary university hospital where diverse patient groups are admitted, such situations can occur, even if it is not a nosocomial outbreak. Therefore, additional tests are required to confirm nosocomial outbreaks. To confirm this, it is essential to investigate whether the pathogens identified in two or more suspected cases are related or share the same strain (1).

Tests to confirm this relatedness can be broadly categorized into DNA-based- and non-DNA-based methods. DNA-based methods can be further divided into amplification-based methods such as multilocus sequencing typing (MLST) and repetitive-PCR genomic fingerprinting method including REP-PCR, BOX-PCR, and ERIC-PCR, and non-amplification-based methods such as pulsed-field gel electrophoresis (PFGE) and whole-genome sequencing (WGS) (2). WGS is considered the gold standard for confirming nosocomial outbreaks because of its ability to utilize the most extensive genetic information and achieve the highest discriminatory power (2). However, utilizing WGS as a routine strain-typing method is challenging owing to its high demands in terms of cost, ease of use, speed, and interpretability (2). MLST, commonly used in clinical settings along with PFGE, involves sequencing seven housekeeping genes to confirm their genetic relatedness at a relatively high resolution (2, 3). However, these methods are not well-suited for species with high genetic diversity because gene recombination can occur relatively easily (3). With the recent widespread adoption of next-generation sequencing, parallel sequencing of numerous genes has become easier. Consequently, there is a growing trend in the use of core-genome MLST (cgMLST), which employs a larger number of genes for typing to overcome the limitations of traditional methods (3).

Rapid detection of nosocomial outbreaks in real-time can reduce patients' hospitalization periods and hospital costs, and most importantly, prevent serious health complications caused by bacterial infections (1). However, DNA-based methods are time-consuming, making them unsuitable for rapid, real-time surveillance (1, 4). Recently, a non-DNA-based typing method called Fourier-transform infrared (FT-IR) spectroscopy, the IR-Biotyper (Bruker GmbH, Bremen, Germany), was introduced. It utilizes the extent to which infrared light is absorbed by various chemical compounds in bacterial cells (1). This advancement allows for real-time screening of outbreaks in hospitals (1).

We investigated suspected cases of a nosocomial outbreaks of vancomycin-resistant *Enterococcus faecium* (VREFM) involving four patients (20 isolates) in the neonatal intensive care unit (NICU) that occurred between December 2022 and January 2023. We planned to cluster the cases using MLST, cgMLST, and IR-Biotyper, and performed a comparative analysis with WGS as the reference.

## MATERIALS AND METHODS

### Bacterial isolates

A total of 30 isolates were used in this study, originating from 14 patients. Twenty VREFM isolates were obtained from clinical specimens such as stool (*n* = 9), urine (*n* = 9), bronchial aspirates (*n* = 1), and gastric juice (*n* = 1) of the four neonates admitted to the NICU at Keimyung University Dongsan Hospital from December 31, 2022 to January 23, 2023, and additional analyses to confirm relatedness were conducted because of the suspicion of a nosocomial outbreak (Table 1). Among the four NICU patients included in this study, patient P2, in particular, had various duplicate samples. P2 was admitted for pneumoperitoneum due to gastric perforation. To check for colonization, stool samples were collected, and urine samples were taken following a routine urinalysis that showed moderate bacteria. Additionally, bronchial aspiration samples were collected for colonization verification, as P2 was receiving respiratory support. As negative controls, we included 10 VREFM isolates obtained from 10 patients in a different ward during a similar timeframe, unrelated to the outbreak (Table 1). All isolates were identified using VITEK MS (bioMérieux SA, Marcy l’Etoile, France), and antimicrobial susceptibility testing was performed using VITEK 2 and VITEK AST-P600 cards. All isolates were stored at −80°C, and subculturing was performed before testing. This study was approved by the Institutional Review Board of Dongsan Medical Center (IRB-2023–11-037).

**TABLE 1:** Information for the isolates tested in this study^*a*^

Isolates	Source patient	Location	Source sample type	Sample collection date	Identification	MLST (sequence type)	cgMLST (complex type)
DS_1	P1	PICU	Rectal swab	20230112	EFM	ST80	CT7742
DS_2	P2^*b*^	NICU	Urine	20230104	EFM	ST17	CT6553
DS_3	P2^*b*^	NICU	Urine	20230105	EFM	ST17	CT6553
DS_4	P2^*b*^	NICU	Urine	20230106	EFM	ST17	CT6553
DS_5	P2^*b*^	NICU	Bronchial aspiration	20230107	EFM	ST17	CT6553
DS_6	P2^*b*^	NICU	Stool-screen	20230105	EFM	ST17	CT6553
DS_7	P2^*b*^	NICU	Stool-screen	20230107	EFM	ST17	CT6553
DS_8	P2^*b*^	NICU	Urine	20230110	EFM	ST17	CT6553
DS_9	P2^*b*^	NICU	Stool-screen	20230112	EFM	ST17	CT6553
DS_10	P2^*b*^	NICU	Urine	20230116	EFM	ST17	CT6553
DS_11	P2^*b*^	NICU	Stool-screen	20230116	EFM	ST17	CT6553
DS_12	P2^*b*^	NICU	Urine	20230119	EFM	ST17	CT6553
DS_13	P3	142	Stool-screen	20230123	EFM	ST262	CT7741
DS_14	P4^*b*^	NICU	Stool-screen	20230105	EFM	ST17	CT6553
DS_15	P4^*b*^	NICU	Stool-screen	20230107	EFM	ST17	CT6553
DS_16	P5^*b*^	NICU	Urine	20221231	EFM	ST17	CT6553
DS_17	P5^*b*^	NICU	Urine	20230102	EFM	ST17	CT6553
DS_18	P5^*b*^	NICU	Urine	20230107	EFM	ST17	CT6553
DS_19	P5^*b*^	NICU	Stool-screen	20230105	EFM	ST17	CT6553
DS_20	P5^*b*^	NICU	Stool-screen	20230107	EFM	ST17	CT6553
DS_21	P6^*b*^	NICU	Gastric	20230113	EFM	ST1421	CT6552
DS_22	P7	CICU	Stool-screen	20230119	EFM	ST1421	CT7743
DS_23	P8	191	Stool-screen	20230125	EFM	ST262	CT7741
DS_24	P9	191	Stool-screen	20230126	EFM	ST17	CT6553
DS_25	P10	191	Stool-screen	20230126	EFM	ST80	CT7744
DS_26	P11	191	Stool-screen	20230125	EFM	ST262	CT7745
DS_27	P12	191	Stool-screen	20230112	EFM	ST80	CT7746
DS_28	P13	191	Stool-screen	20230125	EFM	ST1421	CT6552
DS_29	P14	191	Stool-screen	20230126	EFM	ST17	CT6570
DS_30	P2*^b^*	NICU	Stool-screen	20230123	EFM	ST17	CT6553

^*a*^ Abbreviations: CICU, cardiac intensive care unit; CT, complex type; EFM, *Enterococcus faecium*; NICU, neonatal intensive care unit; PICU, pediatric intensive care unit; ST, sequence type.

^*b*^ Four patients (20 isolates) in neonatal intensive care unit.

### Confirming genetic relatedness

Genomic DNA extraction was carried out from bacterial pellets of the 30 VREFM isolates using the QIAamp DSP DNA Mini Kit (Qiagen, Inc., CA, USA) following the manufacturer’s instructions. The extracted genomic DNA underwent WGS for cgMLST, split-Kmer analysis (SKA), and pairwise comparison using a *de novo* reference (PCDR). Macrogen (Seoul, Korea) performed WGS on an Illumina NovaSeq platform employing 150 bp paired-end sequencing strategies.

The selection and amplification of seven housekeeping genes for multilocus sequence typing (MLST) followed the method described by Homan et al. (5). For determining sequence types (ST), PubMLST (https://pubmlst.org) was used to determine STs (6).

The resulting raw reads of WGS were assembled *de novo* for cgMLST using the Linux versions of SeqSphere+ (v. 9.0.4; Ridom GmbH, Münster, Germany) and SKESA (v. 2.3.0). Following this, *de novo* assembled data were utilized to extract complex types (CT) using the *Enterococcus faecium* (EFM) cgMLST scheme created by de Been et al., as provided by SeqSphere+ (7). The cgMLST scheme included 1423 genes. Using the cgMLST distance matrix provided by SeqSphere+, a neighbor-joining tree was constructed using MEGA 11 (v. 11.0.13).

The SKA and PCDR analyses were conducted following the methods previously reported by Neumann et al. (3). Versions of the packages used for the analysis were SKA v. 1.0, SKESA v. 2.4.0, and Snippy v. 4.6.0. Additionally, the thresholds used for the analysis were set as follows: Snippy with minifrac 0.9, minicov 10, and the rest at the default values. Distance matrices for both PCDR and SKA were created, and neighbor-joining trees were constructed using the MEGA 11. Various SNP distance thresholds have been employed to determine the genetic relatedness between strains. In most studies, researchers have either utilized laboratory-based data or set the threshold using epidemiologically related strains (1, 7–9). Typically, these thresholds have ranged from around 7 to 20 SNPs. In our study, the SNP distance threshold used for WGS clustering was set at 3. This threshold was based on the maximum number of SNPs observed in strains isolated from the same patient, and we classified strains as having relatedness if they had fewer than 3 SNPs between them.

### IR-Biotyper

Thirty isolates were cultured for 24 h on tryptic soy agar supplemented with 5% sheep blood. Each isolate was pre-processed using an IR-Biotyper Kit (Bruker GmbH, Bremen, Germany) according to the manufacturer’s instructions. A loopful (approximately 1 µL) of bacteria was added to 50 µL of 70% ethanol and thoroughly vortexed to ensure complete dissolution. Afterward, 50 µL of distilled water was added and thoroughly mixed to make a total volume of 100 µL. Subsequently, 15 µL aliquots were loaded onto a microtiter plate in three spots each. After drying in a 37°C incubator for approximately 30 min, measurements were taken using an IR-Biotyper with the default settings. The acquired spectra were analyzed using IR-Biotyper software (v. 4.0.3.7334; Bruker GmbH, Bremen, Germany) and OPUS software (v. 8.2.28; Bruker GmbH). The distance matrix was calculated using the Euclidean distance and linkage algorithms, and the UPGMA clustering method was employed. A dendrogram was drawn based on hierarchical clustering analysis, and the clustering cutoff was determined using both the automatically calculated cutoff in the OPUS software and a manually selected cutoff.

### Within-run precision

In the WGS analysis, which included SKA and PCDR, isolates with an SNP distance of zero were considered genetically identical. The proportion of identical isolates classified into the same cluster by the IR-Biotyper was calculated to estimate the within-run precision of the IR-Biotyper.

### Discriminatory power and clustering concordance

Simpson’s Index of Diversity (SID) was used to assess the discriminatory power. The Adjusted Rand Index (ARI) and Adjusted Wallace Index (AWI) were used to confirm clustering concordance. For the calculation of SID, ARI, and AWI, the online tool “Comparing partitions” (http://www.comparingpartitions.info/) was utilized (10).

## RESULTS

### Microbial typing

The ST obtained using MLST and CT obtained using cgMLST from the 30 isolates are presented in Table 1. A total of 20 suspected nosocomial outbreak isolates (DS_2–12, DS_14–21, DS_13) revealed ST17 in 19 isolates and ST1421 in one isolate (DS_21). Similarly, using cgMLST, CT6553 was confirmed in 19 isolates, and CT6552 was identified in one isolate (DS_21). Among the 10 isolates obtained from patients in different wards during a similar timeframe unrelated to the outbreak, two isolates showed ST17 and one isolate exhibited CT6553 (Table 1).

### Dendrograms and phylogenetic trees

**Fig 1:**
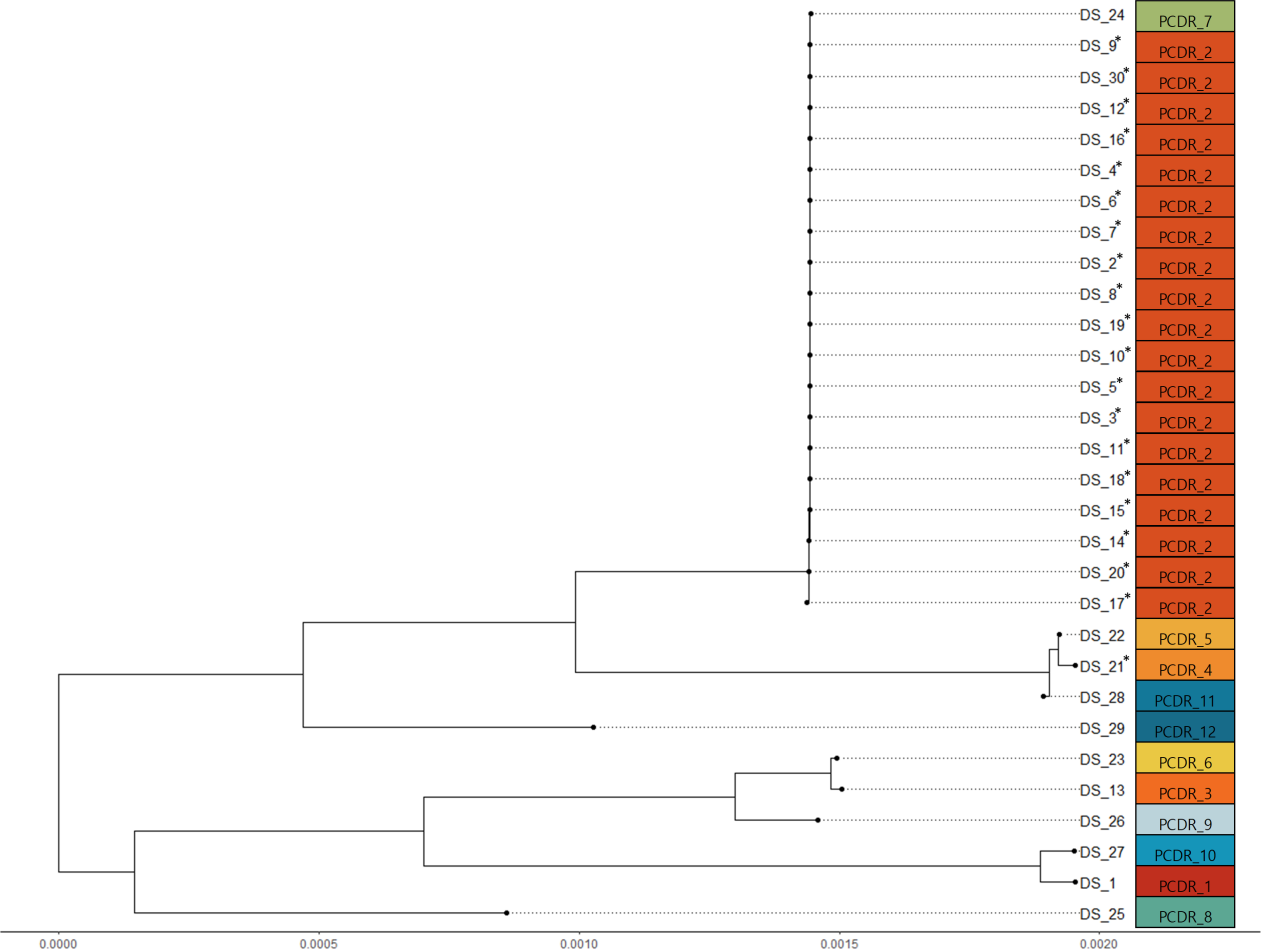
The phylogenetic tree and clustering results obtained from the analysis of WGS data using pairwise comparison using *de novo* references (PCDR). The 20 isolates from 4 patients in the neonatal intensive care unit marked with an asterisk.

The phylogenetic tree and clustering results obtained from the analysis of the WGS data using cgMLST, SKA, and PCDR are presented in Figs. S1 and S2; Fig. 1, respectively. The clustering results obtained from the SKA and PCDR analyses were identical (Fig S1; Fig. 1, respectively). Using WGS, it was determined that out of the 20 isolates from 4 NICU patients, 19 isolates from 3 patients were all genetically identical, except for DS_21 isolated from patient P6. Thus, out of the 20 isolates, 19 isolates exhibited relatedness, confirming a VREFM outbreak in the NICU, while the remaining 1 isolate was unrelated.

**Fig 2:**
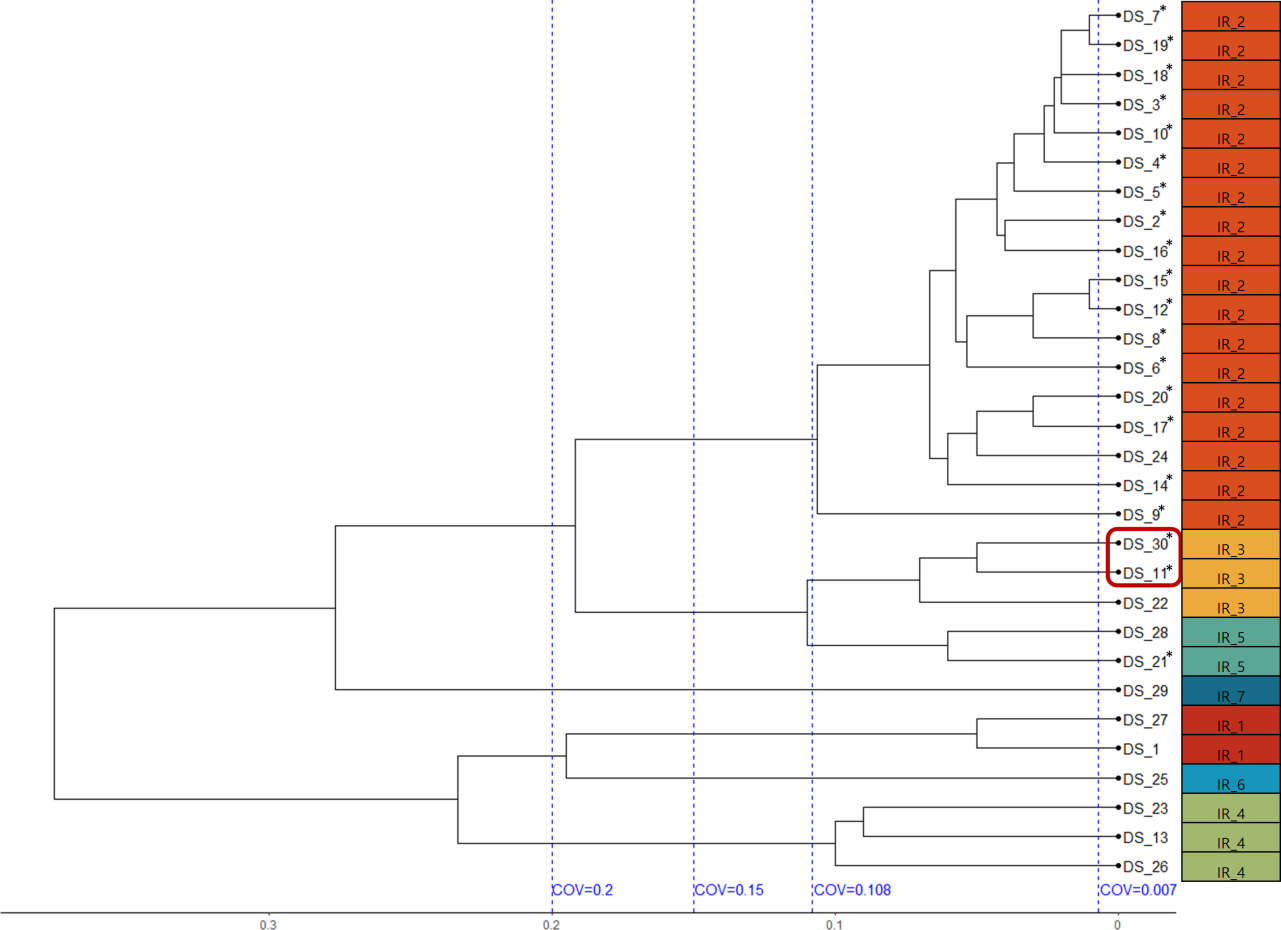
The dendrogram obtained using the IR-Biotyper. The blue vertical dashed lines represent clustering cut-off values (COV). The 20 isolates from 4 patients in the neonatal intensive care unit marked with an asterisk. The red box indicates the two outlier isolates identified in the within-run precision study.

A dendrogram obtained using an IR-Biotyper is shown in Fig. 2. The clustering cutoff automatically calculated using OPUS software was 0.007. The automatically calculated clustering cutoff (0.007), the suggested clustering cutoff provided by the manufacturer for EFM (0.15–0.20), and the final cutoff adopted in this study (0.108) are all depicted in Fig. 2.

### Cut-off determination for clustering using the IR-Biotyper

**Fig 3:**
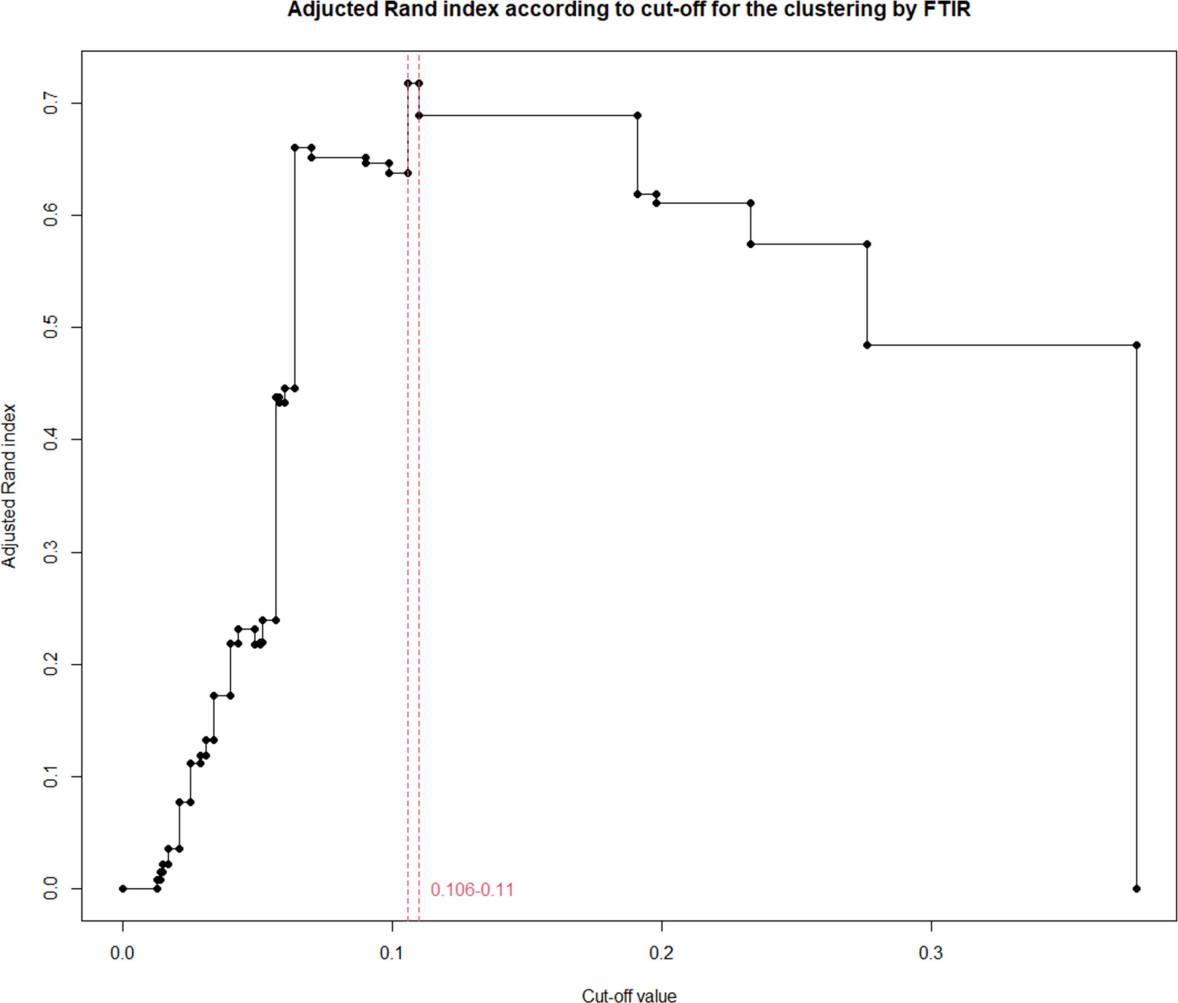
Adjusted Rand index (ARI) according to clustering cut-off for IR-Biotyper. The red vertical dashed line represents the optimal clustering cutoff range with the highest ARI (*n* = 30).

To determine the clustering cut-off in the spectra analyzed by IR-Biotyper, the clustering results of the PCDR analysis were used as a reference to calculate the ARI at various cutoffs (Fig. 3). As identified in Fig. 3, it was observed that a range between 0.106 and 1.111 maintains the same ARI, which was found to be the highest. For the convenience of analysis in this study, a value of 0.108 was used for the clustering cut-off value (Fig. 2); however, it is important to note that using any value within the range of 0.106 to 1.111 would have yielded the same results.

### Within-run precision

Among the 30 isolates, 16 (DS_2–8, 11, DS_14–20, DS_30) showed an SNP distance of 0 in both PCDR and SKA analyses, confirming them as identical isolates. Of the 16 isolates, 14 (87.5%) were confirmed to be classified into the same cluster by IR-Biotyper (clustering cut-off 0.108), whereas two isolates (DS_11 and DS_30) were classified into different clusters (Fig. 2, red box). In contrast, MLST, cgMLST, SKA, and PCDR analyses classified all 16 isolates into the same cluster (Fig. 1; Fig S1 and S2).

### Discriminatory power and clustering concordance among clustering methods

**Fig 4:**
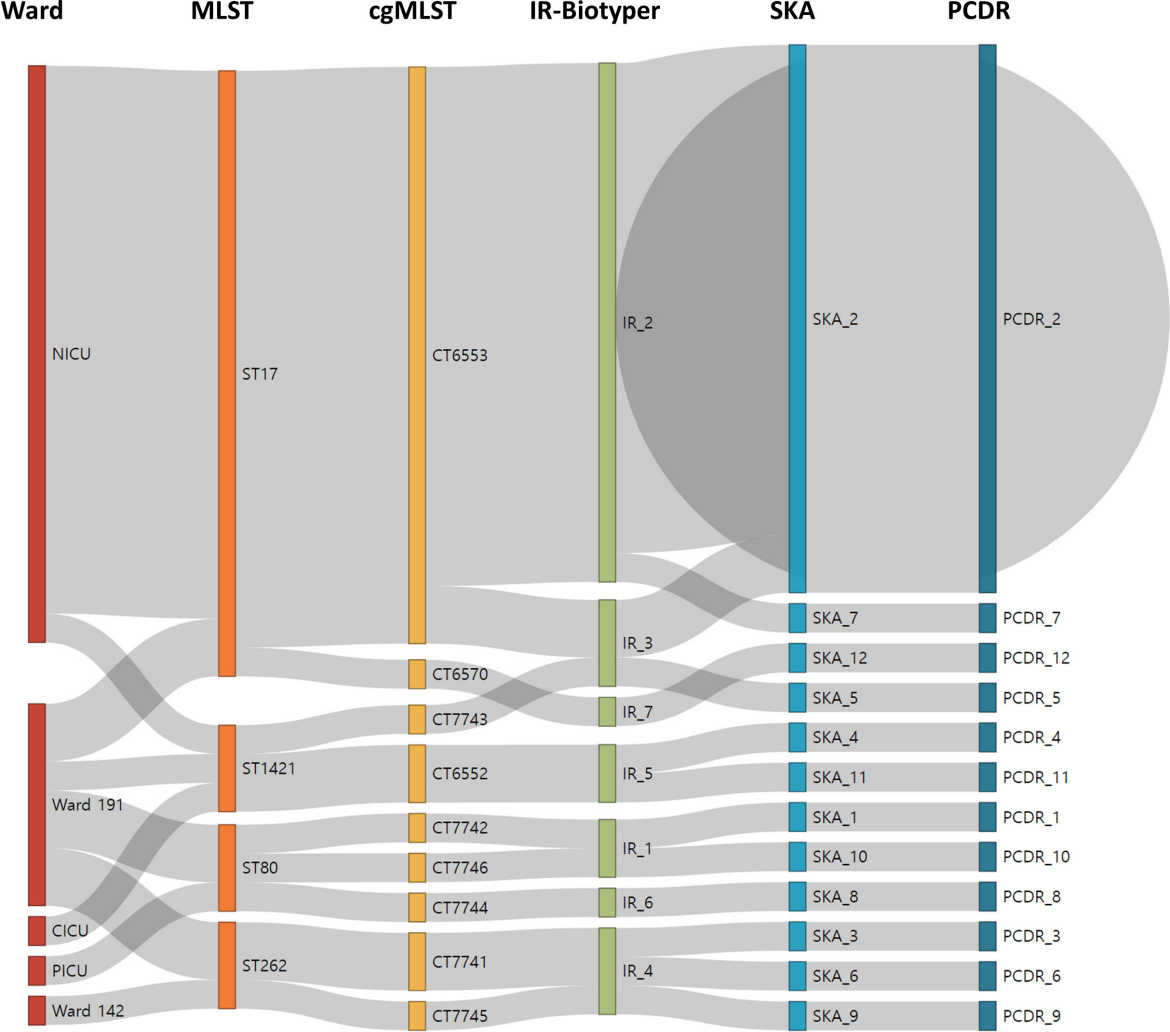
Relationship between methods for clustering (*n* = 30). Abbreviations: PICU, pediatric intensive care unit; NICU, neonatal intensive care unit; CICU, cardiac intensive care unit; EFM, *Enterococcus faecium*; ST, sequence type; CT, complex type.

The correlation between the results of each cluster analysis for the 30 isolates is shown in Fig. 4.

Using the PCDR analysis results as a reference, the SID, ARI, and bidirectional AWI for MLST, cgMLST, SKA analysis, and the IR-Biotyper (clustering cut-off 0.108) are described in Table 2. In terms of ARI, MLST had a higher value than the IR-Biotyper, and AWI (PCDR → MLST) was higher than AWI (PCDR → IR-Biotyper).

**TABLE 2:** Discriminatory power and clustering concordance for each clustering method (*n* = 30)^*a*^

Clustering methods	SID (95% CI)	ARI	AWI (PCDR→Methods)	AWI (Methods→PCDR)
MLST	0.497 (0.296–0.697)	0.78	1	0.639 (0.290–0.988)
cgMLST	0.559 (0.345–0.772)	0.901	1	0.82 (0.533–1.000)
SKA	0.607 (0.396–0.818)	1	1	1
IR-Biotyper (clustering cut-off 0.108)	0.63 (0.443–0.817)	0.718	0.684 (0.328–1.000)	0.754 (0.451–1.000)
PCDR	0.607 (0.396–0.818)			

^*a*^ Abbreviations: ARI, Adjusted Rand Index; AWI, Adjusted Wallace Index; cgMLST, core-genome multilocus sequencing typing; MLST, multilocus sequencing typing; PCDR, pairwise comparison using de novo references; SID, Simpson’s Index of Diversity; SKA, split-kit analysis.

On the other hand, excluding the two outlier isolates (DS_11 and DS_30) identified in the study for within-run precision, both ARI and bidirectional AWI calculations favored the IR-Biotyper over MLST (Table 3).

**TABLE 3:** Discriminatory power and clustering concordance for each clustering method (excluding the two outliers, *n* = 28)^*a*^

Clustering methods	Sid (95% CI)	ARI	AWI (PCDR→Methods)	AWI (Methods→PCDR)
MLST	0.524 (0.322–0.726)	0.764	1	0.618 (0.264–0.973)
cgMLST	0.59 (0.375–0.805)	0.894	1	0.809 (0.511–1.000)
SKA	0.64 (0.429–0.851)	1	1	1
IR-Biotyper (clustering cut-off 0.108)	0.582 (0.375–0.789)	0.878	1	0.783 (0.490–1.000)
PCDR	0.64 (0.429–0.851)			

^*a*^ Abbreviations: ARI, Adjusted Rand Index; AWI, Adjusted Wallace Index; cgMLST, core-genome multilocus sequencing typing; MLST, multilocus sequencing typing; PCDR, pairwise comparison using de novo references; SID, Simpson’s Index of Diversity; SKA, split-kit analysis.

## DISCUSSION

Vancomycin-resistant Enterococci (VRE) are commonly identified as nosocomial pathogens linked to various infections, including urinary tract infections, surgical site infections, and bloodstream infections (11). Recent results from KORGLASS phase I (2017–2019), indicate a steady increase in the proportion of VRE in bloodstream infections caused by EFM, reaching 38.1%. This increase has been consistent from 2017 to 2019 (12). Notably, samples corresponding to healthcare-associated infections constitute a significant burden, accounting for approximately 43.0% of nosocomial outbreaks. EFM is known for its high recombinogenic rate and genetically diverse, making it insufficient for accurate genetic relatedness studies using widely used clinical methods such as MLST or PFGE (3). In this study, we aimed to compare the clustering results obtained using MLST, cgMLST, and the IR-Biotyper with those from WGS as tools for assessing nosocomial outbreaks of VREFM. The goal of the study was to determine whether the use of the IR-Biotyper in practice is sufficiently helpful for detecting nosocomial outbreaks of VREFM.

FT-IR, introduced in the 1950s, utilizes the extent of infrared light absorption by various chemical compounds in bacterial cells to determine bacterial relatedness (4). The unique fingerprints generated reflect cellular components, such as nucleic acids, proteins, lipids, and carbohydrates. Therefore, each bacterium possessed highly specific infrared absorption characteristics, allowing for the typing of bacteria at the subspecies level using FT-IR (4). In 2017, Bruker launched IR-Biotyper, a system based on FT-IR technology, focusing on providing an integrated system for the subspecies-level typing of bacteria (2). The effectiveness of typing and clustering analysis using the IR-Biotyper has been reported for a variety of microorganisms, including Gram-negative bacilli such as *Klebsiella pneumoniae*, *Escherichia coli*, *Enterobacter cloacae*, *Pseudomonas aeruginosa*, and *Acinetobacter baumannii* (1, 2, 4, 8, 13–17), and Gram-positive cocci such as *Staphylococcus aureus*, *Enterococcus* species (1, 11, 18), *Legionella pneumophila* (19), non-tuberculosis Mycobacteria (20, 21), *Neisseria gonorrhoeae* (22), and *Candida parapsilosis* (23).

Two limitations were reported in the clustering analysis of the IR-Biotyper; the following two limitations have been reported to date. The clustering cutoff of the IR-Biotyper is crucial in determining the cluster results, and ultimately, different cutoffs must be used for each strain. However, the first limitation of IR-Biotyper is the lack of a standardized clustering cutoff owing to insufficient research conducted for each strain. Indeed, for *Candida parapsilosis*, there are reports of using a higher cut-off of 0.9 or above (23). In contrast, Scheier et al.’s study on EFM employed and validated a relatively lower cut-off range of 0.14–0.17 (11), showcasing the variability in cut-off values based on different microbial strains. Even within the same microbial strain, such as *K. pneumoniae*, the reported cutoff values vary. Some studies targeting *K. pneumoniae* have utilized a relatively higher cut-off range of 0.15–0.3 (2, 14), while other research has suggested lower cut-offs with satisfactory concordance (4).

The second limitation of the IR-Biotyper lies in the potential impact of various factors during the pre-analysis and pre-processing steps on the test results and the lack of well-established standardization in this regard. While most testing processes have been standardized with the launch of the IR-Biotyper, some studies suggest that differences in factors, such as the type of media to obtain colonies, incubation time for cultivation, and other variables, can impact the results (2, 11). Therefore, to ensure consistent test results, it is essential not only to set clustering cut-offs for each strain but also to standardize the pre-processing steps, including the culture medium used and incubation time. Moreover, to enhance standardization, it is essential to include adequate details about the clustering cutoff value and the pre-processing steps employed when reporting the results of studies utilizing the IR-Biotyper.

In this study, colonies obtained after 24 h of incubation on tryptic soy agar supplemented with 5% sheep blood were pre-processed according to the manufacturer’s guidelines. To determine the optimal clustering cut-off, we calculated cut-off-specific ARI values using the PCDR analysis results from WGS as a reference. A cutoff range of 0.106–0.11, which yielded the highest ARI, was identified as the optimal cutoff range.

Subsequently, we adopted 0.108 as the optimal clustering cutoff for our analysis for the convenience of analysis in this study. The manufacturer of the IR-Biotyper suggests a cut-off range for strain-level typing, and for EFM, they propose a range of 0.15–0.2 (Fig. 3). Additionally, the IR-Biotyper system is equipped with various algorithms to automatically determine the cut-off values. In our study, the automatically calculated cutoff value was 0.007. Therefore, the optimal clustering cut-off (0.108) under the conditions used in this study was relatively lower than the cut-off suggested by the manufacturer (0.15–0.2) and higher than the automatically calculated cut-off (0.007) (Fig. 2). This was also lower than the optimal clustering cutoff adopted in a previous study by Scheier et al. for EFM, which was in the range of 0.14–0.17 (11). Scheier et al. used the species-level discrimination ability and clustering concordance between replicates using the same samples to determine the optimal cut-off. Hence, the optimal cut-off identified in our study, aimed at maximizing clustering with WGS, might differ from the findings of Scheier et al.

Utilizing the determined optimal clustering cutoff, MLST, cgMLST, and IR-Biotyper were analyzed and compared with the clustering results obtained from WGS to confirm nosocomial outbreaks. The analysis of 30 isolates revealed that, using PCDR (0.607, 95% CI 0.396–0.818) as the reference, both MLST (0.497, 95% CI 0.296–0.697) and cgMLST (0.559, 95% CI 0.345–0.772) demonstrated lower discriminatory power, whereas IR-Biotyper (0.63, 95% CI 0.443–0.817) exhibited higher discriminatory power (Table 2). MLST and cgMLST, which target a smaller number of housekeeping genes than WGS, may have a lower resolution, potentially leading to underclassification. The higher discriminatory power observed with the IR-Biotyper than with WGS may be explained by the overclassification of the IR-Biotyper. However, it is important to note that overclassification does not necessarily imply misclassification. This is because even with the same genotype, microorganisms can exhibit phenotypic differences (4).

Interestingly, in the within-run precision analysis using genetically identical isolates with an SNP distance of 0 in the WGS analysis, two isolates (DS_11 and DS_30) were classified into different clusters, indicating lower precision compared with other testing methods. In particular, isolates DS_11 and DS_30, along with 10 other isolates (DS_2–10, DS_12), were isolated from the same patient (P2) (Table 1). These 12 isolates were obtained from the same patient (P2) and confirmed to have the same genotype by WGS. Therefore, they can be classified as identical. However, the IR-Biotyper classified them into two separate clusters. These two isolates, although genetically identical to isolates DS_2–10 and DS_12, differ as indicated in Table 1; they originate from different sample types and were collected at different times. In the case of P2, the longest duration of sample collection among the patients was enrolled, especially for isolates DS_11 and DS_30, which were cultured from the last two stool samples collected. This fact suggests that the discordant results for DS_11 and DS_30 could be attributed to differences in the type of source samples and the timing of collection, namely the difference in storage duration. There are several reports suggesting that the surrounding environment and storage conditions of microbial strains can impact their RNA expression (24, 25). The influence of the type of source samples and storage conditions of bacterial strains on the clustering results of the IR-Biotyper is an area that warrants further investigation.

Excluding these two isolates (DS_11 and DS_30), the analysis of the remaining 28 isolates indicated that the discriminatory power of the IR-Biotyper was lower than that of the PCDR (Table 3). Therefore, based on these results, it is challenging to assert that the IR-Biotyper has a higher discriminatory power than WGS. Although it exhibited lower within-run precision, it can be considered to have higher discriminatory power than MLST.

In evaluating the relatedness of strains suspected in nosocomial outbreaks, both discriminatory power and precision are crucial. Low discriminatory power may lead to misinterpreting non-outbreak situations as outbreaks, while low precision can result in imprecise results, creating errors in underclassification or overclassification. While the low discriminatory power of MLST, due to its technical limitations, may not be easily improved except by adopting a larger number of target genes, the low within-run precision of the IR-Biotyper could be ameliorated by controlling various pre-processing steps.

In the analysis of clustering concordance with PCDR using 30 isolates, the IR-Biotyper (0.718) exhibited a lower ARI than MLST (0.78), indicating the lowest ARI in this study (Table 2). However, when comparing 28 isolates, excluding the DS_11 and DS_30 isolates, the IR-Biotyper (0.878) showed a higher ARI than the MLST (0.764) (Table 3).

Assuming the use of the IR-Biotyper for the purpose of nosocomial screening, if the same cluster is identified using the IR-Biotyper, confirmation will be performed with a method with higher discriminatory power, such as WGS. Methods with higher discriminatory power are less likely to cluster strains into the same group compared to those with lower power. AWI (WGS → IR-Biotyper) refers to the probability that strains clustered together in WGS will also be clustered together in the IR-Biotyper, hence it is bound to be high. However, a low AWI (WGS → IR-Biotyper) could indicate that the performance of the IR-Biotyper, including its clustering concordance and precision, is insufficient for clinical outbreak screening. Conversely, AWI (IR-Biotyper → WGS) is naturally low due to the lower discriminatory power of the IR-Biotyper. However, too low an AWI (IR-Biotyper → WGS) may lead to increased false clustering by the IR-Biotyper, necessitating more unnecessary WGS tests. Therefore, Teng et al. set a criterion for using the IR-Biotyper in real-time screening of nosocomial outbreaks as AWI (WGS → IR-Biotyper) of over 0.95 and AWI (IR-Biotyper → WGS) of over 0.50 (1). In this study, analyzing 28 isolates excluding DS_11 and DS_30, the AWI (WGS → IR-Biotyper) of IR-Biotyper was 1.0, and the AWI (IR-Biotyper → WGS) was 0.783, satisfying both criteria. Additionally, from the perspective of real-time screening for nosocomial outbreaks, the IR-Biotyper has advantages over other genetic microbial strain typing methods due to its shorter turnaround time and lower cost (4). If strains can be typed in real-time using the IR-Biotyper, without the need for storage, imprecision could potentially be improved. However, for effective utilization as a real-time screening tool for nosocomial outbreaks, the low within-run precision of the IR-Biotyper needs to be improved.

This study confirmed that three out of four infants who developed VREFM infection in the NICU between December 31, 2022 and January 23, 2023, were identified as nosocomial outbreaks using an IR-Biotyper. Additionally, this suggests that the IR-Biotyper, showing superior clustering concordance with WGS compared to the widely used MLST, could be sufficiently utilized as a screening test for detecting VREFM-related nosocomial outbreaks.

Our study had several limitations. First, we did not evaluate the impact of growth media or incubation time on IR-Biotyper. Second, we did not assess the between-run precision. Third, we could not analyze the reasons for the overclassification of isolates DS_11 and DS_30 compared with WGS. Additional studies with larger numbers of isolates are needed to address these limitations and provide more comprehensive insights.

## Data Availability

All raw sequencing data generated were submitted to the Sequence Read Archive (SRA) under the BioProject accession number PRJNA1049039.

## SUPPLEMENTAL MATERIAL

Supplemental figures spectrum.04119-23-s0001.docx Fig. S1 and S2.

ASM does not own the copyrights to Supplemental Material that may be linked to, or accessed through, an article. The authors have granted ASM a non-exclusive, world-wide license to publish the Supplemental Material files. Please contact the corresponding author directly for reuse.
