# Supplementary material for: Comparative analysis of IR-Biotyper, MLST, cgMLST, and WGS for clustering of vancomycin-resistant Enterococcus faecium in a neonatal intensive care unit
Source: Microbiol Spectr. 2024 Mar 5;12(4):e04119-23. doi: 10.1128/spectrum.04119-23 (PMC10986520; doi:10.1128/spectrum.04119-23)

Supplemental Data

**S1 Figure.** The phylogenetic tree and clustering results obtained from the analysis of WGS data using SKA.

**
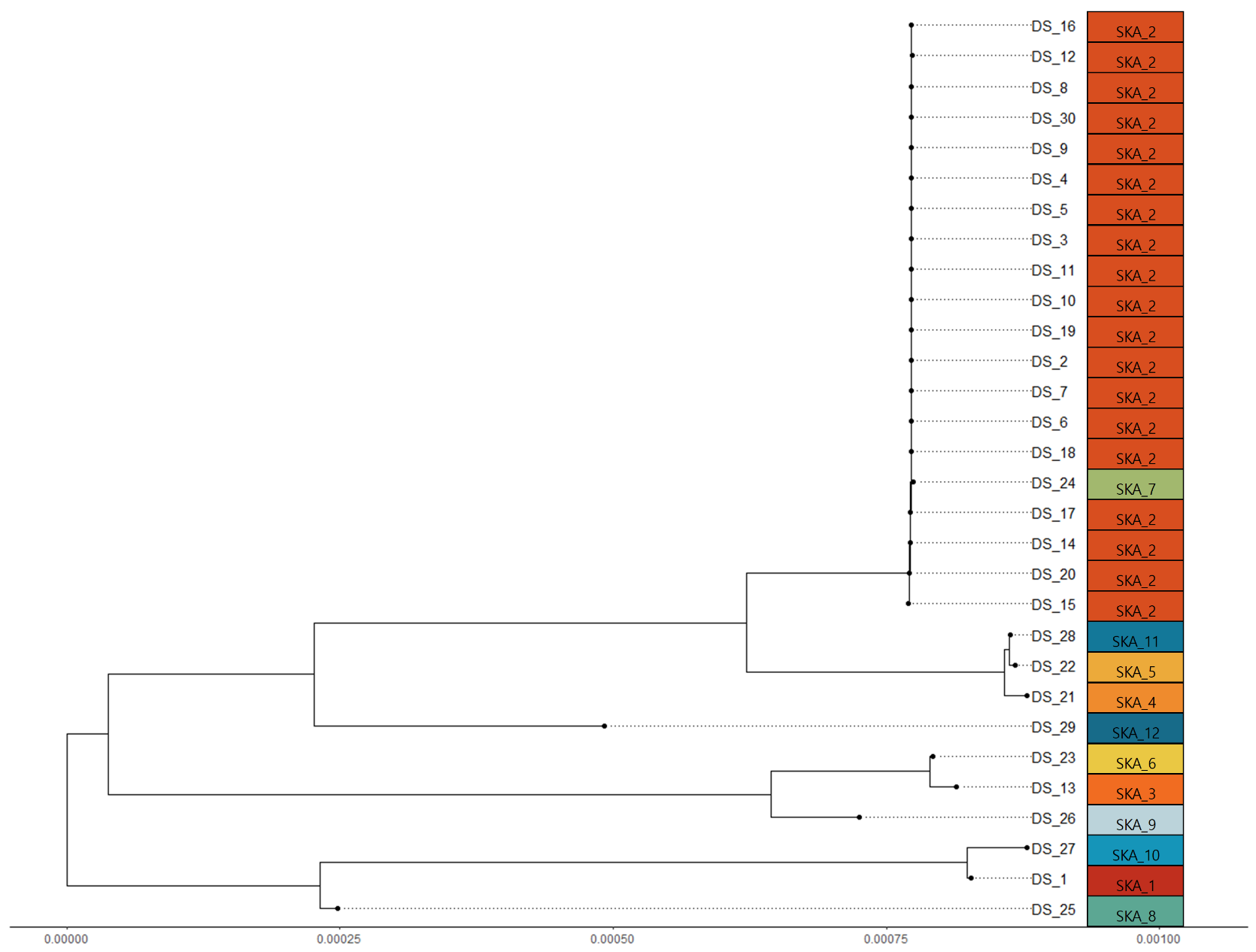
**

**S2 Figure**. The phylogenetic tree and clustering results obtained from the analysis of WGS data using cgMLST.


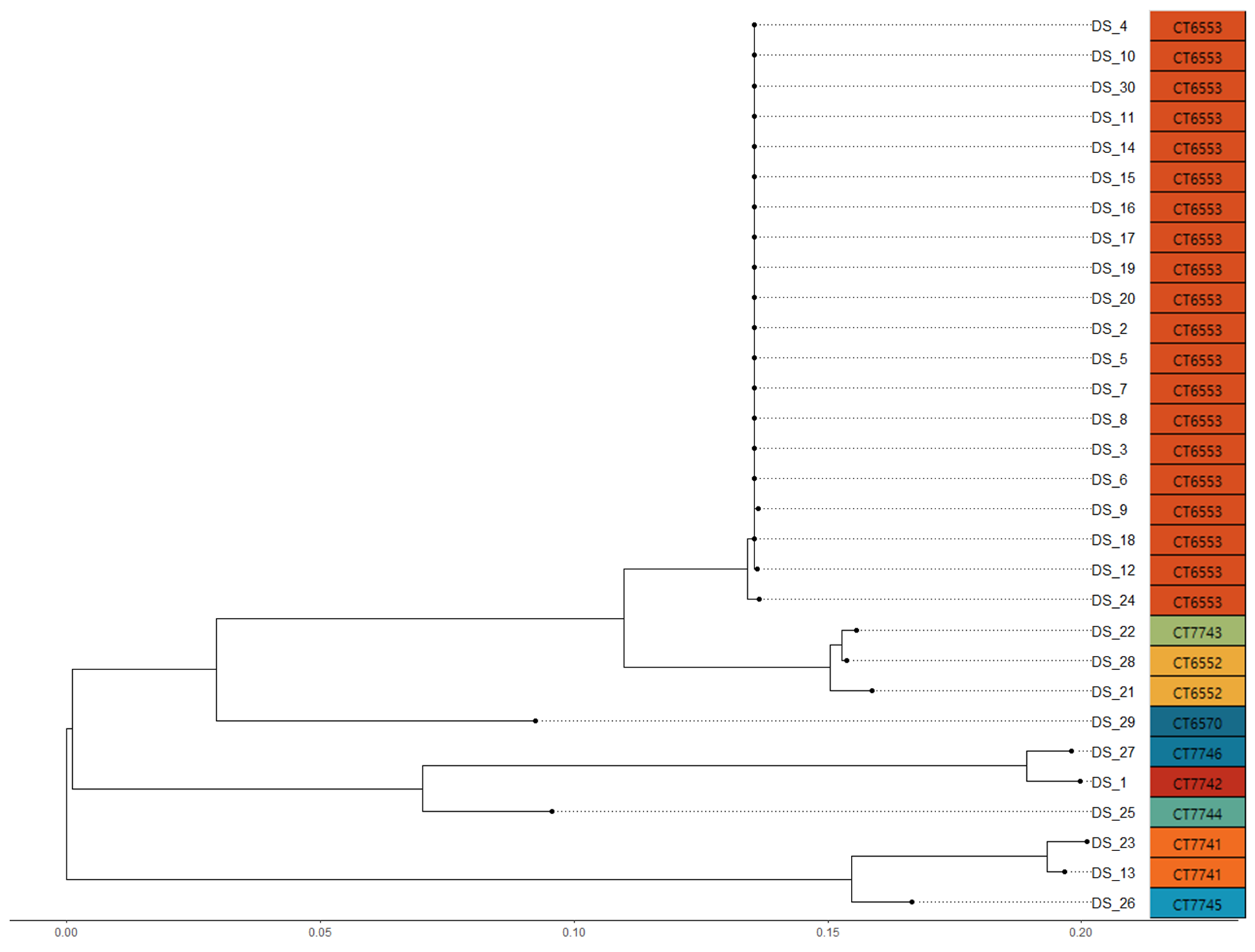

Supplement: Supplemental figures — Fig. S1 and S2. [file spectrum.04119-23-s0001.docx]
